# Vitamin D Deficiency Reduces Vascular Reactivity of Coronary Arterioles in Male Rats

**DOI:** 10.3390/cimb43010007

**Published:** 2021-05-07

**Authors:** Zoltán Fontányi, Réka Eszter Sziva, Éva Pál, Leila Hadjadj, Anna Monori-Kiss, Eszter Mária Horváth, Rita Benkő, Attila Magyar, Andrea Heinzlmann, Zoltán Benyó, György L. Nádasy, Gabriella Masszi, Szabolcs Várbíró

**Affiliations:** 1 Department of Obstetrics and Gynaecology, Semmelweis University, Üllői Street 78/a, 1082 Budapest, Hungary; fontanyi.zoltan@med.semmelweis-univ.hu (Z.F.); varbiro.szabolcs@med.semmelweis-univ.hu (S.V.); 2 Department of Physiology, Semmelweis University, Tűzoltó Street 37-47, 1094 Budapest, Hungary; horvath.eszter@med.semmelweis-univ.hu (E.M.H.); benko.rita@med.semmelweis-univ.hu (R.B.); nadasy.gyorgy@med.semmelweis-univ.hu (G.L.N.); 3 Department of Translational Medicine, Semmelweis University, Üllői Street 78/a, 1082 Budapest, Hungary; pal.eva@med.semmelweis-univ.hu (É.P.); leila.hadjadj@gmail.com (L.H.); anna.monorikiss@gmail.com (A.M.-K.); benyo.zoltan@med.semmelweis-univ.hu (Z.B.); 4 Department of Anatomy, Histology and Embriology, Semmelweis University, Tűzoltó Street 58, 1094 Budapest, Hungary; magyar.attila@med.semmelweis-univ.hu; 5 Department of Anatomy and Histology, University of Veterinary Medicine, István Street 2, 1078 Budapest, Hungary; hmannandrea@gmail.com; 6 Department of Internal Medicine, National Institute of Mental Health, Neurology and Neurosurgery, Lehel Street 59-61, 1135 Budapest, Hungary; gabriellamasszi@gmail.com

**Keywords:** vitamin D deficiency, male, rat model, cardiovascular disease, pharmacological reactivity, thromboxane, estradiol, testosterone

## Abstract

Background: Vitamin D deficiency (VDD) may be considered an independent cardiovascular (CV) risk factor, and it is well known that CV risk is higher in males. Our goal was to investigate the pharmacological reactivity and receptor expression of intramural coronary artery segments of male rats in cases of different vitamin D supply. Methods: Four-week-old male Wistar rats were divided into a control group (*n* = 11) with optimal vitamin D supply (300 IU/kgbw/day) and a VDD group (*n* = 11, <0.5 IU/kgbw/day). After 8 weeks of treatment, intramural coronary artery segments were microprepared, their pharmacological reactivity was examined by in vitro microangiometry, and their receptor expression was investigated by immunohistochemistry. Results: Thromboxane A_2_ (TXA_2_)-agonist induced reduced vasoconstriction, testosterone (T) and 17-β-estradiol (E2) relaxations were significantly decreased, a significant decrease in thromboxane receptor (TP) expression was shown, and the reduction in estrogen receptor-α (ERα) expression was on the border of significance in the VDD group. Conclusions: VD-deficient male coronary arteries showed deteriorated pharmacological reactivity to TXA_2_ and sexual steroids (E2, T). Insufficient vasoconstrictor capacity was accompanied by decreased TP receptor expression, and vasodilator impairments were mainly functional. The decrease in vasoconstrictor and vasodilator responses results in narrowed adaptational range of coronaries, causing inadequate coronary perfusion that might contribute to the increased CV risk in VDD.

## 1. Introduction

Several conflicting opinions exist on the link between vitamin D and cardiovascular diseases (CVDs). Although we still do not have any certain knowledge about the exact beneficial effect of vitamin D on CVDs, it seems that vitamin D deficiency (VDD) may be an independent cardiovascular risk factor associated with a higher risk of cardiovascular diseases, events, and mortality [1,2,3].

Vitamin D deficiency can be diagnosed when the serum 25-hydroxyvitamin D (25(OH)D) level is below 20 ng/mL (or <50 nmol/L) [4,5]. Based on this limit value, more than 40% of the world’s population is vitamin D deficient [5]. Vitamin D exerts its effects on both nuclear and membrane vitamin D receptors (VDRn and VDRm); while VDRn belongs to the classical nuclear receptor family and is located in the nucleus, VDRm can be found on the cell surface, plasma membrane, and perinuclear area [6,7]. A vitamin-D-deficient state promotes not only endothelial dysfunction through influence on the structure and function of endothelial cells, but also vascular smooth muscle cell proliferation [8].

The sex difference in cardiovascular risk has been well-known since the Framingham Study [9,10,11,12]; men have relatively higher risk than women do, so reproductive-aged women are protected from these diseases. The most common cause of cardiovascular morbidity and mortality is coronary artery diseases (CADs) in both sexes, although the clinical appearance of the illnesses may differ [13]. In several acute CADs, such as myocardial infarction, angina pectoris, and sudden cardiac death, pro-thrombogenic factors (platelets and thromboxane A_2_ production) play an important role [14].

Thromboxane A_2_ (TXA_2_) is one of the main prostanoids that is generated not only by activated platelets but also by endothelial cells and vascular smooth muscle cells [15], synthetized by thromboxane-A synthase through the prostanoid pathway. TXA_2_ mediates paracrine and autocrine effects through its thromboxane A_2_ receptor (TP, which is also available for other prostanoids [15]) and causes mainly platelet activation, degranulation, aggregation, and, in circulus vitiosus, further TXA_2_ production. Besides this, it is an effective vasoconstrictor and plays a role in angiogenesis and inflammation [16]. TXA_2_ binds to TP receptor, which is coupled to G-protein (G_q/11_ or G_12/13_) and activates phospholipase C (PLC); through this pathway, it finally increases the intracellular calcium concentration, which results in vasoconstriction [15]. With hydrolysis, TXA_2_ is converted quickly into thromboxane B_2_ (TXB_2_), a biologically inactive metabolite.

Sexual steroids have potential roles in cardiovascular sex-related variance; testosterone (T) and 17-β-estradiol (E2) are the two most potent sexual steroids (T in men and E2 in women), and both can cause genomic and rapid, non-genomic vasodilation via several different mechanisms and pathways, mostly in the appropriate sex [17,18].

Estrogens have two classical nuclear receptors, estrogen receptors α and β (ERα, ERβ), and membrane-bound G-protein-coupled receptor 30 or G-protein-coupled estrogen receptor 1 (GPR30 or GPER1) [17,19]. By reducing endothelial dysfunction, causing vasodilation through a nitric oxide (NO)-mediated pathway, and decreasing vasoconstriction and vascular smooth muscle cell proliferation, estrogens have anti-hypertensive effects and play an important role in cardiovascular protection in women of reproductive age [17]. Estrogen-induced beneficial vascular responses are mainly mediated by endothelial ERα [20].

Androgens also have nuclear and ‘membrane-bound or other’ receptors. Besides the classical nuclear androgen receptor (AR), numerous receptors, signal transduction pathways, and ion channels have been investigated, which may have possible roles in androgen-induced vasodilation: the PLC, G-protein coupled receptor C6A (GPRC6A), oxoeicosanoid receptor 1 (OXER1), L dual oxidase 1 (DUOX1), Zinc transporter protein 9 (ZIP9), L-type voltage-dependent calcium-channel (LVDCC), voltage-dependent potassium channel (K_v_), small- and large-conductance calcium-activated potassium channels (SK_Ca_, BK_Ca_), PI3K/Akt signaling pathway, modulation of cAMP and cGMP levels, mitochondrial procaspase 3 and 8, NADPH oxidase, and transient receptor potential cation channel family members (TRPM8, TRPV4) [18,21]. Estrogens and androgens may affect platelet functions via the sex difference that was described in the effect of antithrombotic drugs [22].

However, the possible relationship of a vitamin-D-deficient state and pharmacological vascular responses of small vessels in the male sex is a less-researched area. Our aim was to investigate possible pharmacological reactivity changes of intramural resistance coronary artery segments in response to vasoconstrictor and several vasodilator agents in a vitamin-D-deficient state, and to further reveal the possible details and initial steps of increased cardiovascular risk in men.

## 2. Materials and Methods

### 2.1. Animals

Twenty-two 4-week-old, 100–140 g weighted male *Wistar* rats (Semmelweis University, Charles River, Budapest, Hungary) were involved in this 8-week-long experiment. Rats were housed 4–5 together in a constant light–dark (12:12 h) cycle and controlled temperature (22 ± 1 °C) and humidity (56%) and were supplied with tap water and with normal or vitamin-D-deficient rat chow ad libitum.

### 2.2. Treatment and Experimental Protocol

Rats were distributed into two groups randomly, and we induced different vitamin D status in the animals as follows: From the first week, the control group (*n* = 11) received normal vitamin-D-containing conventional rat chow (containing 1000 IU/kg of vitamin D, SM Rat/mouse normal diet S8106-S011, Ssniff Spezialdiäten GmbH, Soest, Germany) constantly and vitamin D supplementation per os (Vigantol, 20.000 IU/mL, Merck/Serono, Mumbai, India). Taken together, the daily vitamin D intake in the control group was approximately 300 IU/kgbw, providing optimal vitamin D supply.

The vitamin-D-deficient group (*n* = 11) received <0.5 IU/kgbw/day vitamin D for eight weeks to model vitamin D deficiency (VDD) status (rat chow containing <5 IU vitamin D, EF Rat/mouse VitD-free diet E15312-24, Ssniff Spezialdiäten GmbH, Soest, Germany; measured average daily chow intake: 0.1 kg/bwkg chow). To validate our treatment protocol, serum 25-hydroxyvitamin D levels were measured from blood samples and found to be 5 times lower in the VDD group compared to control animals at the 8th week of treatment, as our team published earlier [23]; this indicates the effectiveness of our treatment.

After 8 weeks, animals were sacrificed. Under general surgical anesthesia (Nembutal, 45 mg/kg b.w., i. p. Ceva-Phylaxia, Budapest, Hungary) and after perfusion via the carotid artery with heparinized Krebs-Ringer solution to wash out all blood from the vascular system, the chest was opened and the heart was removed, then the heart weights were measured. The intramural coronary arteriole was isolated (terminal branch of the left anterior descending/LAD coronary artery about in vivo 150–200 μm outer diameter at preparation) from the left ventricular muscle tissue under a stereomicroscope (Wild M3Z, Heerbrugg, Switzerland) [24]. Coronary arteriole segments with 150–200 micrometer outer diameter (length of about 2 mm) were cut off for pressure microarteriography, and further sections of the arteries with surrounding ventricular tissue were cut off for immunohistochemical examinations.

### 2.3. Pressure Microarteriography of Coronary Arterioles

The excised coronary arteriole segments were placed into an organ chamber (Experimetria Ltd., Budapest, Hungary) filled with normal Krebs-Ringer solution (nKR), cannulated at both ends with microcannulas, and each was extended to its in vivo length. The composition of the nKR solution (in mM/L) was: NaCl 119; KCl 4.7; NaH_2_PO_4_ 1.2; MgSO_4_ 1.17; NaHCO_3_ 24; CaCl_2_ 2.5; glucose 5.5; and EDTA 0.034. The chamber was placed on the stage of a microscope (Leica, Wetzlar, Germany). Pressure-servo pumps (Living Systems, St. Albans Burlington, VT, USA) were connected to both cannulas, and the arterioles were pressurized to 50 mmHg intraluminal pressure. The segments were allowed to equilibrate for 30 min at this pressure in nKR bubbled with a 5% CO_2_, 20% O_2_, and 75% N_2_ gas mixture, and the temperature was kept at 37 °C during the whole measurement period. After the arterioles were allowed to equilibrate at 50 mmHg intraluminal pressure for 10 min, cumulatively increasing concentrations of 17-β-estradiol (Tocris Bio-Techne, Bristol, UK) were added to the chamber (10^−8^–10^−5^ mol/L, 8 min incubation for each step). After washing the segments with nKR and allowing them to equilibrate at 50 mmHg intraluminal pressure for 10 min, the segments were exposed to 10^−8^ and 10^−6^ mol/L testosterone (Sigma-Aldrich, Darmstadt, Germany) with 5 min of incubation for both doses. Thereafter, with repeated washing with nKR and equilibration at 50 mmHg intraluminal pressure for 10 min, a cumulative dose–response curve with insulin (Actrapid penfill 100 IU/mL, Novo Nordisk, Bagsværd, Denmark) was constructed (30, 100, 300, and 600 IU/L). After 8 min incubation with each concentration, the vessel chamber was washed out and equilibrated at 50 mmHg intraluminal pressure for 10 min with nKR again. Then, 10^−6^ mol/L U46619, a stable thromboxane A_2_ receptor agonist, in a concentration causing maximal vasoconstriction (Tocris Bio-Techne, Bristol, UK) was administered. After this, the arterioles were allowed to equilibrate at 50 mmHg intraluminal pressure for 10 min. The endothelial relaxation capacity of arteriole segments was tested by cumulative application of adenosine (Adenocor, Sanofi-Aventis, Madrid, Spain) (10^−9^–10^−6^ mol/L, 3 min incubation for each step at 50 mmHg intraluminal pressure). Finally, the fully relaxed diameter of vessels was measured in calcium-free Krebs solution; its composition was as follows (in mM/L): NaCl 92; KCl 4.7; NaH_2_PO_4_ 1.18; MgCl_2_ 20; MgSO_4_ 1.17; NaHCO_3_ 24; glucose 5.5; EGTA 2; and EDTA 0.025. All compounds were purchased from Sigma-Aldrich (St. Louis, MO, USA). Pictures were taken during the measurement by a digital histological video camera (Leica DFC 320, Leica, Wetzlar, Germany) connected to the microscope. The outer and inner diameters/D_o_ and D_i_ of the vessels were measured on the magnified pictures of the arterioles in ImageJ image analysis software (Image J 1.50b, National Institutes of Health, Bethesda, MD, USA). For the calibration, an Etalon micrometer (Wild, Heerbrugg, Switzerland) was used.

### 2.4. Calculations

From the inner and outer diameters, the following vessel characteristics were calculated:

• Inner radius/R_i_ (μm):

(1) $$R_{i}=\frac{D_{i}}{2},$$

• Outer radius/R_o_ (μm):

(2) $$R_{o}=\frac{D_{o}}{2},$$

• Myogenic tone (%):

(3) $$\mathrm{Myogenic}\;\mathrm{tone}\;\left(\%\right)=\frac{R_{o\;\mathrm{Ca}\;-\;\mathrm{free}}-{\;R}_{o\;\mathrm{nKR}}}{R_{o\;\mathrm{Ca}\;-\;\mathrm{free}}}\;\times \;100,$$

• TXA_2_-agonist-induced constriction (%):

(4) $${\mathrm{TXA}}_{2}-\;\mathrm{constriction}\;\left(\%\right)=\frac{R_{o\;\mathrm{Ca}\;-\;\mathrm{free}}-{\;R}_{o\;\mathrm{TXA}2}}{R_{o\;\mathrm{Ca}\;-\;\mathrm{free}}}\;\times \;100,$$

• 17-β-estradiol-induced relaxation (%):

(5) $$E2-\;\mathrm{relaxation}\;\left(\%\right)=\frac{R_{o\;E2}-{\;R}_{o\;\mathrm{nKR}}}{R_{\mathrm{nKR}}}\;\times \;100,$$

• Testosterone-induced relaxation (%):

(6) $$T\;-\;\mathrm{relaxation}\;\left(\%\right)=\frac{R_{o\;T}-{\;R}_{o\;\mathrm{nKR}}}{R_{o\;\mathrm{nKR}}}\;\times \;100,$$

• Adenosine-induced relaxation (%):

(7) $$\mathrm{ADE}\;-\;\mathrm{relaxation}\;\left(\%\right)=\frac{R_{o\;\mathrm{ADE}}-{\;R}_{o\;\mathrm{TXA}2}}{R_{o\;\mathrm{TXA}2}}\;\times \;100,$$

• Insulin-induced relaxation (%):

(8) $$\mathrm{INZ}\;-\;\mathrm{relaxation}\;\left(\%\right)=\frac{R_{o\;\mathrm{INZ}}-{\;R}_{o\;\mathrm{nKR}}}{R_{o\;\mathrm{nKR}}}\;\times \;100$$

### 2.5. Immunohistochemistry of Coronary Arterioles

Coronary arteriole segments were freshly fixed with 4% formaldehyde. They were embedded in paraffin, sectioned, and mounted on a glass slide. Native sections were used for immunohistochemical (IHC) investigations. IHC stainings were performed against vitamin D receptor (VDR), endothelial nitric oxide synthase (eNOS), estrogen receptor-α (ERα), thromboxane receptor (TP) and androgen receptor (AR). Mouse monoclonal anti-VDR (1:200, Santa Cruz Biotechnology, sc-13133, Dallas, TX, USA), anti-eNOS (1:50, Abcam, ab76198, Cambridge, UK) and rabbit polyclonal anti-ERα (1:100, Merck/Sigma-Aldrich, 06-935, St. Louis, MO, USA), anti-TP (1:50, MyBioSource, MBS2032166, San Diego, CA, USA), and anti-AR (1:100, Abcam, ab74272, Cambridge, UK) antibodies were applied. Secondary labeling was achieved by using a horseradish peroxidase (HRP)-labeled horse anti-rabbit and anti-mouse IgG polymer detection kit (Vector Laboratories, MP-7401, MP-7402, Burlingame, CA, USA, 30–40 min). A brown-colored 3-3′-diamino-benzidine peroxidase HRP substrate kit (Vector Laboratories, SK-4100, Burlingame, CA, USA) was used to visualize the specific antigen labeling, and blue-colored Hematoxylin QS nucleus stain (Vector Laboratories, H-3404-100, Burlingame, CA, USA) was used as counterstaining.

The immunostained sections were photographed with a microscope-coupled video camera (Zeiss Axio Imager.A1 with Zeiss AxioCam MRc5 CCD, Carl Zeiss, Jena, Germany or Nikon Eclipse Ni-U, 933584 with Nikon DS-Ri2 camera and NIS Elements BR image software, Nikon Corporation, Tokio, Japan). The area percentages of different parts of the vessel wall (tunica intima, media, or whole part) were measured from the digitized pictures using ImageJ image analysis software.

### 2.6. Statistical Analysis

Statistical analysis was performed with the help of GraphPad Prism 7.0 (GraphPad Software, San Diego, CA, USA) statistical software. After checking the Kolmogorov–Smirnov, D’Agostino and Pearson omnibus, and Shapiro–Wilk normality tests, in case of data with a normal distribution, we used the parametric unpaired T-test with F-test; data with a non-normal distribution were analyzed using the non-parametric Mann–Whitney U-test. Repeated-measures variance analysis (ANOVA) with Bonferroni’s post hoc test was used in case of pharmaceutical agent doses. Because of the few applied doses, we decided to present our results in dose–response curves instead of nonlinear regression. For all statistical analyses, *p* < 0.05 was considered statistically significant. All values are expressed as the Mean ± SEM or Median [IQR]. Significance symbols: *: *p* < 0.05; **: *p* < 0.01.

## 3. Results

### 3.1. Heart Weight

As our workgroup published previously, physiological, hormonal, and glucose-metabolism parameters; systolic and diastolic blood pressures; and the body weight of the rats measured did not differ between the two groups at the end of the experiment [23,25]. There was no significant difference between heart weights either (in Mean ± SEM: 1.41 ± 0.051 g and 1.55 ± 0.048 g for the control and VDD groups, respectively, n.s.), indicating that the vitamin-D-deficient diet, at least within 8 weeks, did not affect this cardiovascular parameter.

### 3.2. Coronary Arteriole Morphology and Function

**Figure 1:**
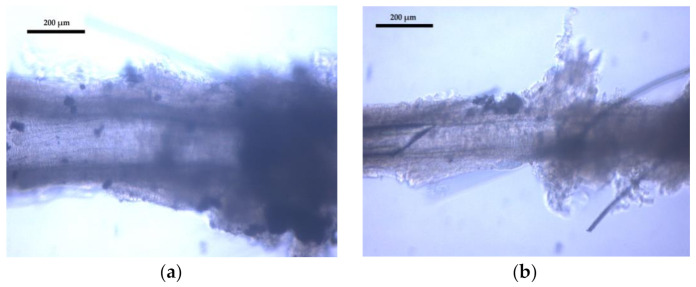
Representative images of intramural coronary artery segments from the control (**a**) and vitamin-D-deficient (**b**) groups in normal Krebs-Ringer solution at 50 mmHg intraluminal pressure. Scale bar: 200 μm.

Significant differences were observed in inner radii: the VDD group had significantly lower inner radius than the control group (*p* < 0.01). In contrast, the outer radii of the coronary arteriole segments measured in passive circumstances seemed to be smaller but this difference did not reach the level of statistical significance in the two groups; the same was the case for the myogenic tone (Table 1, Figure 1).

### 3.3. Constriction Capacity of Coronary Arterioles: Thromboxane-A_2_-Induced Contraction

**Figure 2:**
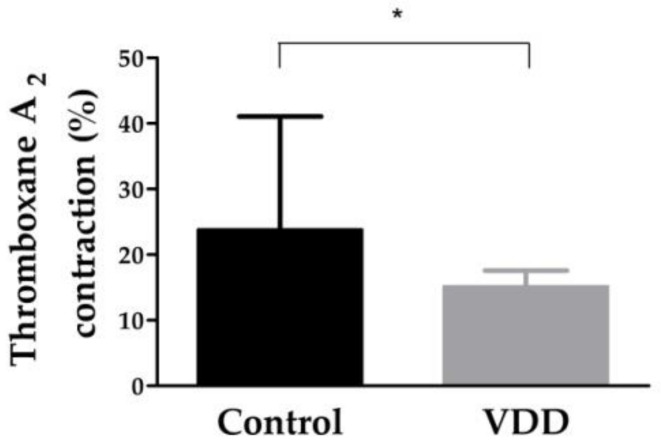
Thromboxane-A_2_-induced contraction of coronary arterioles. (*n* = 7 − 7) Calculated data are presented for 50 mmHg intraluminal pressure. Mann–Whitney U test, Median [IQR], *: *p* < 0.05.

Thromboxane-A_2_-induced contraction was significantly (*p* < 0.05) decreased in the VDD group compared to the control group (Figure 2).

### 3.4. Relaxation Ability of Coronary Arterioles: 17-β-Estradiol-, Testosterone-, Adenosine-, and Insulin-Induced Relaxation

**Figure 3:**
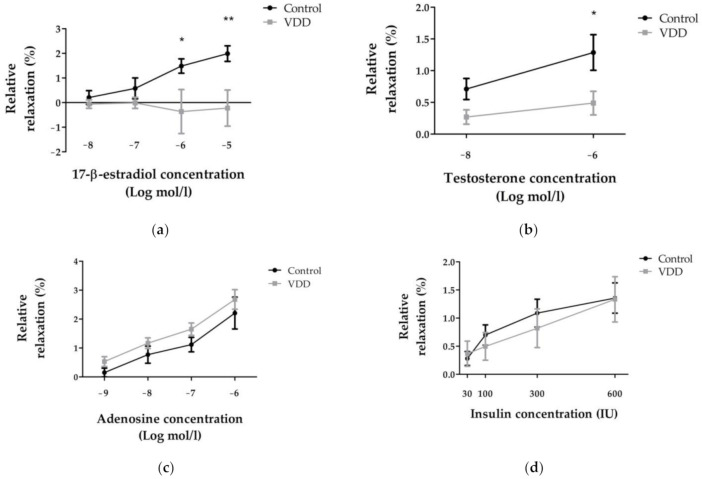
Relaxations of coronary arterioles induced by the administration of 17-β-estradiol ((**a**); %, *n* = 10 − 9), testosterone ((**b**); %, *n* = 10 − 8), and adenosine ((**c**); %, *n* = 8 − 10) in normal Krebs-Ringer solution in the presence of U46619 TXA2-agonist at 50 mmHg intraluminal pressure, and insulin-induced relaxation ((**d**); %, *n* = 8 − 7) in normal Krebs-Ringer solution. Repeated-measures ANOVA, Bonferroni. Mean ± SEM, *: *p* < 0.05; **: *p* < 0.01.

While the control group relaxed in response to 17-β-estradiol, the vitamin-D-deficient group showed significantly lower relaxation (*p* < 0.05 and *p* < 0.01) and mild contraction at higher doses (Figure 3a).

Testosterone-induced vasodilation was significantly lower (*p* < 0.05) in the VDD group than in the control group (Figure 3b).

Adenosine-induced relaxation that was measured after thromboxane-A_2_-agonist contraction did not differ between the two groups (Figure 3c).

Insulin vasodilation was similar in both groups, though there were some variations between the shapes of the curves (Figure 3d).

### 3.5. Immunohistochemical Stainings: TP, ERα, AR, eNOS, and VDR

**Figure 4:**
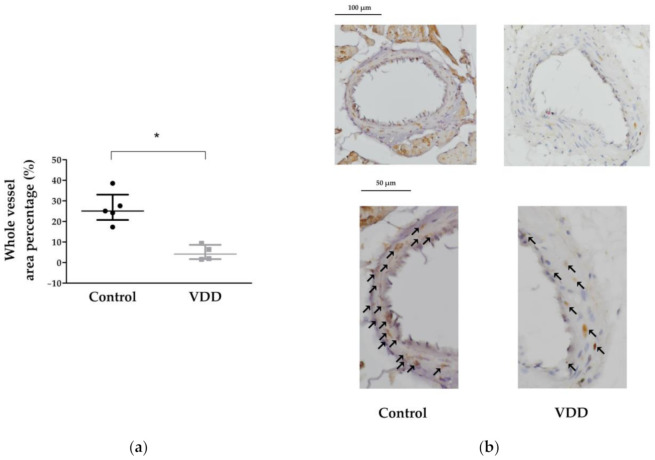
Results of thromboxane receptor immunohistochemical staining. (**a**) Percentage of the coronary arteriole cross-sectional area positively stained with anti-TP antibodies. Mann–Whitney U-test. Median [IQR], *n* = 5 − 4. *: *p* < 0.05; (**b**) Representative images of anti-TP-stained tissue sections of male rat coronary arteriole segments. Brown color indicates the TP-positive areas in both groups: in the control group, the whole vessel (endothelium and vascular smooth muscle cells) was stained, as well as the surrounding ventricular tissue, while in the VDD group, the brown color is much less pronounced. Scale bars: 100 and 50 μm. Black arrows indicate positively stained cells.

Thromboxane receptor (TP) expression was significantly reduced in the vitamin-D-deficient group, which is consistent with the result of decreased TXA_2_-induced vasoconstriction in this group (Figure 4a,b).

**Figure 5:**
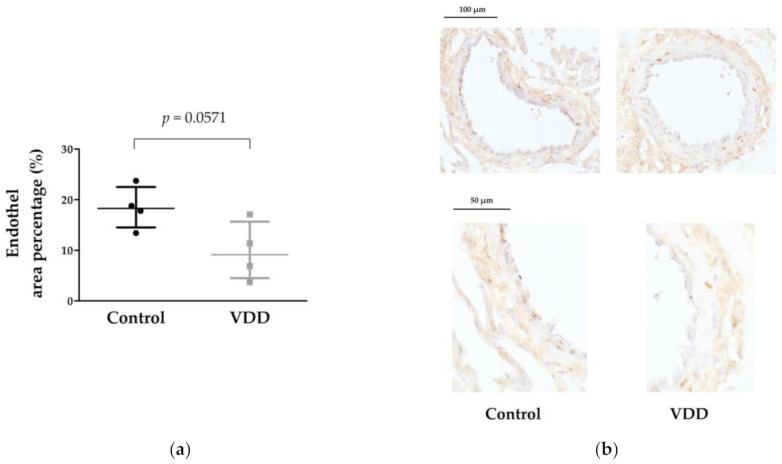
Results of anti-estrogen-receptor-α immunohistochemical staining. (**a**) Percentage of the coronary arteriole cross-sectional endothelial area positively stained with anti-ERα antibodies. Mann–Whitney U-test. Median [IQR], *n* = 4 − 4. *p* = 0.0571; (**b**) Representative images of anti-ERα-stained tissue sections of male rat coronary arteriole segments. Brown color indicates the ERα-positive areas in both groups: ERα expression is similar in the tunica media layer; however, in the VDD group the staining intensity of the endothelium is less visible. Scale bars, 100 and 50 μm.

The estrogen-receptor-α expression was lower in the endothelial layer of the VDD group’s coronary arteriole segments; the difference was on the border of statistical significance (*p* = 0.0571) (Figure 5a,b).

The androgen receptor expression showed no difference between the control and vitamin-D-deficient groups (positively stained endothelial area%: 28.33 [12.06–56.03] and 23.40 [9.146–32.07] for the control and VDD groups, respectively, n.s.).

The investigation of endothelial NO-synthase expression showed that the vitamin-D-deficient diet did not affect the expression of that enzyme in the intimal layer of the vessels (positively stained endothelial area%: 13.87 [10.30–33.28] and 15.48 [7.10–20.94] for the control and VDD groups, respectively, n.s.).

There was also no difference in vitamin D receptor expression between the groups (positively stained whole vessel area%: 23.25 [14.90–29.69] and 26.18 [17.64–37.68] for the control and VDD groups, respectively, n.s.).

## 4. Discussion

To our knowledge, this is the first study finding significant alterations in the reaction of small intramural coronary arteriole segments to a vasoconstrictor and sexual steroids (estrogen and androgen) due to a vitamin-D-deficient state. Moreover, a part of the identified damages appeared at the molecular level, and the results of receptor expression examinations are in accordance with the identified pharmacological vasoreactivity impairments. Our main findings are that a vitamin-D-deficient state caused reduced inner radii, decreased thromboxane A_2_ agonist vasoconstriction, reduced thromboxane receptor expression, and diminished 17-β-estradiol and testosterone vasodilator capacity of the intramural coronary artery of male rats.

Reduced inner radii with unchanged outer radii may indicate vessel wall reorganization causing lumen narrowing, while the myogenic tone of the arteriole segments was not affected. Our workgroup detected inward eutrophic remodeling that can be considered as pre-hypertensive alteration in these small coronary arterioles in response to vitamin D deficiency [25].

Vasoconstrictor capacity, investigated by assessing thromboxane A_2_ agonist, significantly decreased in the coronary arteries of vitamin-D-deficient male rats, as did the expression of thromboxane A_2_ receptor (TP). Sex difference in thromboxane response has previously been observed: isolated coronary arteriole segments’ TXA_2_-induced constriction was found to be significantly higher in male than in female animals [26]. Vitamin D may have a regulatory role in prostanoid pathways. The active form of vitamin D (1,25-dihidroxyvitamin D or 1,25(OH)_2_D) significantly decreased the cyclooxigenase-2 mRNA and protein expression and caused a significant dose-dependent increase in the prostaglandin-catabolizing 15-hydroxyprostaglandin dehydrogenase enzyme expression. Furthermore, vitamin D significantly reduced prostaglandin E2 (PGE_2_) secretion and repressed PGE_2_ receptor isoform, EP2, and prostaglandin F_2α_ receptor FP expression in human prostate cancer and prostatic epithelial cell lines [27,28,29]. In an endotoxin shock male mouse model, 1-α-hydroxyvitamin D, 1,25(OH)_2_D, and 24,25-dihydroxyvitamin D reduced the plasma TXB_2_ levels, suggesting a role of vitamin D in thromboxane A_2_ production [30]. On the contrary, aortic rings of 1,25(OH)_2_D-treated spontaneously hypertensive (SH) and Wistar-Kyoto (WKY) rats showed no difference in U46619-induced vasoconstriction and in thromboxane synthase gene and protein expression [31]; additionally, exogenous 1,25(OH)_2_D administration to isolated, de-endothelized aortic rings from SH and WKY rats did not cause a change in U46619-induced endothelium-independent contraction [32]. A human study found that after cardioplegia/reperfusion, the human coronary microvasculature showed significantly decreased TXA_2_-induced vasoconstriction compared to that before procedures, while the protein level and gene expression of thromboxane synthase and thromboxane A_2_ receptor did not differ in atrial tissue [33]. Vitamin-D-deficient pregnant female Wistar rats had higher placental mRNA levels and lower protein levels of phospholipase A2 and cyclooxigenase-2 compared to the control group, while serum thromboxane B_2_ levels were similar between the groups [34]. Moreover, the VDD group had higher liver and plasma arachidonic acid levels than the control one [35]. Similar results to ours were found in the coronary arterioles of vitamin-D-deficient female animals, TXA_2_ tone massively reduced in both VDD groups independently of their testosterone treatment [36]. Currently, our knowledge is inadequate about the possible link between vitamin D, vitamin D deficiency, and thromboxane A_2_ in the male sex; this needs further detailed investigations.

Considering the relaxation capacities of the coronary arteriole segments from vitamin-D-deficient male animals, 17-β-estradiol- and testosterone-induced vasodilation showed a significant reduction, while relaxation in response to insulin and adenosine showed no difference compared to the control group. Testosterone administration produced significant dose-dependent vasorelaxation on the intrinsic tone of male Wistar rats’ left and right coronary artery segments [37]. In the cerebral arteries of vitamin-D-deficient male rats, significantly enhanced testosterone-induced tone (opposite calculation to relaxation) and reduced AR expression were observed [38], which may indicate that vitamin D deficiency affects the receptor expression of distinct vascular regions differently. It is likely that nuclear and membrane receptor activation, effects on the vascular endothelium, and ion channel activation or inhibition may contribute to the rapid, non-genomic androgen-induced relaxation. The following hypothesis may explain our results: the altered testosterone-induced relaxation and still unchanged nuclear AR expression may suggest the involvement of ‘membrane-bound or other’ receptors, signal transduction pathways, and ion channels in the identified functional damage. Additionally, it is probable that both endothelium-dependent and endothelium-independent, direct vascular smooth muscle cell activation pathways are present [18], but the exact mechanisms and the detailed cardiovascular effects of androgens are still unclear.

In the male sex, the main sexual steroids are naturally the androgens. Thus, this could be a reason why in our experiment, in response to vitamin D deficiency, the estrogen-induced relaxation was altered first and was accompanied by initial changes in estrogen receptor-α expression. 17-β-estradiol-induced relaxation on perfused isolated hearts was significantly lower in male normotensive, control Wistar rats compared to female ones [39]; this sex difference could not be observed in spontaneous hypertensive animals [40], and orchiectomy caused a significant decrease in the E2 relaxation response of males compared to that in ovariectomized female animals [41], although castration did not damage this response fully.

One of the most important functions of endothelial NO-synthase (eNOS) is developing endothelium-dependent relaxation through NO production [42], and both estrogens and androgens play a role in this NO-mediated vasodilator pathway [17,21]. In our experiment, eNOS expression did not show any difference between the groups, which may indicate the deterioration of other, vascular-endothelium- and NO-independent relaxation pathways of sexual steroids due to vitamin D deficiency.

According to our results, in the male sex, vitamin D deficiency may induce alteration first in the vasoconstrictor characteristics of coronary arteries, because both functional and molecular changes developed clearly, while in terms of vasodilator reactivity, sexual-steroid-related impairments appeared mostly in vessel functionality. Other vasodilator pathways, such as adenosine, the most important vasodilator for coronary arteries, and insulin, were not yet affected. It seems that androgen–vitamin D effects in males are less connected than estrogen–vitamin D actions in females in association with carbohydrate–insulin metabolism. It has been proven that insulin relaxation is not altered in male coronary vessels due to vitamin D deficiency, while in vitamin-D-deficient females, insulin-induced relaxation is significantly decreased both in small coronary segments and in large, aortic rings [43,44].

The association of vitamin D levels with cardiovascular diseases has been widely investigated [1,45]. Vitamin D receptors (VDRs) are expressed in endothelial and vascular smooth muscle cells of the vasculature [46], and vitamin D may play a role in the regulation of endothelial functions [47]. Vitamin D deficiency can be associated with endothelial dysfunction [46] and may have sex differences. In a human clinical cross-sectional study, the endothelial function of elderly (and, according to the data, vitamin-D-insufficient) individuals was investigated, and in women, but not in men, vitamin D levels were significantly associated with endothelium-independent vasodilation [48].

During our experiment, VDR expression was similar in the control and vitamin-D-deficient groups. A significant elevation in VDR expression was observed in coronary artery segments of vitamin-D-deficient female rats [43]. Vitamin-D-dependent and -independent actions of VDR have been described, and vice versa, VDR-dependent and -independent vitamin D actions have been distinguished [49].

The beneficial effects of vitamin D supplementation on cardiovascular health have not been proved in large randomized, controlled, and follow-up clinical studies (e.g., the ‘VITAL’, ‘DO-HEALTH’, and ‘VIDA’ studies) [50,51,52]. However, it is important to highlight that these trials involved not only vitamin-D-deficient and -insufficient participants (25(OH)D < 30 ng/mL or <75 nmol/L), but also vitamin-D-sufficient (25(OH)D > 30 ng/mL or >75 nmol/L) participants. Thus, it is necessary to investigate adequately the effects of vitamin D supplementation in only vitamin-D-deficient and in vitamin-D-insufficient target groups in both sexes and different ages (infants, children, adolescents, adults, and the elderly).

Despite the controversies, vitamin D deficiency can be considered an independent cardiovascular risk factor [2]. A population-based study and meta-analysis of several studies showed that increased risk for myocardial infarction, ischemic heart disease, and early death are associated with decreasing 25(OH)D level [53]. Vitamin-D-insufficient middle-aged men had significantly lower coronary flow reserve than a vitamin-D-sufficient group [54]. Combined testosterone and vitamin D deficiencies in a coronary angiography-referred study population of older men showed a significant association with all-cause, cardiovascular, and non-cardiovascular mortality compared to men without any hormone deficiency [55].

In summary, according to our results, vitamin-D-deficient male coronary arteries showed damaged pharmacological reactivity to TXA_2_ and different sexual steroid hormones (E2, T). The most important adenosine vasodilator pathway in coronary arteries was still intact. Insufficient vasoconstrictor capacity occurred in decreased TP receptor expression, while the vasodilator alterations were mainly functional. The possible background of these differences may be the involvement of other, non-genomic endothelium-independent vasodilator pathways of sexual steroids. In case of a decrease in both vasoconstrictor and vasodilator responses, the adaptational range of the intramural coronaries is narrowed, which causes inadequate coronary perfusion and deteriorates the heart’s blood supply.

## 5. Conclusions

To our knowledge, this is the first experiment that has investigated the vascular reactivity of small intramural coronary artery segments from vitamin-D-deficient male rats in response to thromboxane, 17-β-estradiol, testosterone, adenosine, and insulin. We found significant alterations in thromboxane vasoconstriction and estrogen and androgen sexual-steroid-induced relaxations, which have already been shown partially on the level of receptor expression. These alterations narrow the pharmacological adaptational range of these small vessels, which leads to the deterioration of the heart’s perfusion. In addition to chronic hypoperfusion, their current adaptation abilities also worsen. The described changes might contribute to the increased cardiovascular risk in vitamin D deficiency.

## Figures and Tables

**Table 1:** Morphological and functional parameters of coronary arterioles. Inner and outer radii were measured in calcium-free solution; myogenic tone was calculated as described below for 50 mmHg intraluminal pressure. Unpaired T-test or Mann–Whitney U-test. Data are expressed as Mean ± SEM or Median [IQR], ^1^ *n* = 8 − 8 and ^2^ *n* = 6 − 6 in each group, **: *p* < 0.01.

Parameters/Groups	Control	VDD
Inner radii (μm) ^1^:	89.06 ± 5.23	61.17 ± 5.81 **
Outer radii (μm) ^1^:	131.6 ± 8.49	108.3 ± 7.55
Myogenic tone (%) ^2^:	5.14 [1.30–18.41]	2.44 [1.36–4.18]
